# Maternal RSV vaccination generates high-affinity antibodies that efficiently transfer to infants, providing enhanced passive immunity

**DOI:** 10.1038/s41467-026-72659-3

**Published:** 2026-05-08

**Authors:** Dongxiao Liu, Olivia Posadas, Ashish K. Mishra, Mohamed M. Mire, Mindy Pike, Janet A. Englund, Alisa Kachikis, Surender Khurana

**Affiliations:** 1 Division of Viral Products, Center for Biologics Evaluation and Research (CBER), FDA, Silver Spring, MD USA; 2 Department of Obstetrics and Gynecology, University of Washington, Seattle, WA USA; 3 Department of Pediatrics, University of Washington, and Seattle Children’s Research Institute, Seattle, WA USA

**Keywords:** Protein vaccines, Protein vaccines, Paediatric research, Outcomes research

## Abstract

Respiratory syncytial virus (RSV) remains the leading cause of hospitalization in young children. A bivalent pre-fusion (preF) protein RSV vaccine was licensed for pregnant persons in 2023 (Abrysvo, Pfizer), but the impact of RSV maternal immunization on maternal-fetal immunity has not been well characterized. We analyze neutralizing antibody responses, binding antibodies, and antibody affinity maturation in 58 unvaccinated and 49 vaccinated pregnant individuals and their infants. Maternal RSV vaccination induces robust neutralizing antibodies against both RSV-A2 and RSV-B1 strains, with geometric mean titers 8-fold and 13.4-fold higher, respectively, in vaccinated versus unvaccinated participants. Vaccination significantly enhances binding antibodies to RSV-preF protein (5.2-fold higher) and antibody affinity maturation (3.7-fold higher). High-quality RSV-specific antibodies are efficiently transferred across the placenta. However, early preterm infants showed reduced antibody transfer efficiency compared with full-term infants. These findings demonstrate that maternal RSV vaccination generates high-quality, affinity-matured transferable antibodies that provide passive immunity to infants.

## Abstract (web summary)

The authors report that maternal RSV vaccination induces robust affinity-matured neutralizing antibodies in mothers and infants. Antibody transfer increased after 36 weeks, supporting current vaccination recommendations, and suggesting that early vaccination may benefit preterm-risk pregnancies.

## Introduction

Respiratory syncytial virus (RSV) is one of the most significant respiratory viral pathogens affecting infants and young children globally, contributing to approximately 33 million RSV-associated infections in children younger than 5 years and 50,000-100,000 deaths annually worldwide^1^. RSV is the most common cause of hospitalization in infants during the first year of life^2,3^. The vulnerability of infants to severe RSV disease is attributed to their still maturing immune systems, smaller airways, and limited maternally derived immunity^2^. Preterm infants are at particularly elevated risk of morbidity and mortality due to RSV. RSV, in the Pneumoviridae family, has two major antigenic subtypes, A and B, among which A2 and B1 are the representative reference strains that are used in current vaccines and immunological studies for evaluation of vaccines and therapeutics. The virus encodes several key structural proteins, with surface fusion protein (F) and attachment glycoprotein (G) serving as primary targets for neutralizing antibodies and vaccine development^4^.

The development of RSV prevention measures, including monoclonal antibody products and vaccines, has been marked by both challenges and recent breakthroughs. The past formalin-inactivated RSV (FI-RSV) vaccine in the 1960s led to enhanced disease severity upon natural infection, and highlighted the complexity of RSV-specific immunology and the critical importance of inducing appropriate immune responses^5^. Recent advances in the characterization of the RSV fusion protein have led to the development of long-lasting pre-F protein monoclonal antibodies and stabilized pre-fusion F (preF) protein-based vaccines, which have shown superior immunogenicity compared with post-fusion F proteins^6,7^. Maternal immunization has emerged as a promising strategy for protecting young infants during the first months of life. The concept leverages the natural process of maternal antibody transfer across the placenta, primarily through neonatal Fc receptor (FcRn)-mediated active transport of immunoglobulin G (IgG) antibodies^8,9^. The success of maternal vaccination programs against pathogens, including tetanus, influenza virus, and pertussis, has provided a framework for using maternal immunization to provide RSV disease prevention for infants^9^.

The recent licensure of the maternal Pfizer RSV vaccine (Abrysvo) represents a significant milestone in RSV prevention^10^. Abrysvo is a bivalent vaccine containing stabilized prefusion F proteins from both RSV-A and RSV-B subtypes, designed to induce high levels of neutralizing antibodies that can be transferred to the fetus. Clinical trials have demonstrated efficacy in preventing severe RSV disease in infants, leading to regulatory approval and CDC recommendations for administration between 32–36 weeks’ gestation in the United States, though timing recommendations vary internationally^11,12^. This approach to RSV prevention has been implemented in the US, UK, and multiple other countries. However, detailed characterization of the immune responses generated by maternal RSV vaccination, including the quality and quantity of antibodies transferred to cord blood, remains under active investigation. Understanding the immune profile following maternal RSV vaccination is crucial for optimizing vaccination strategies and predicting protective efficacy.

Therefore, in this study, we performed a comprehensive evaluation of maternal and cord blood immune profiles following RSV vaccination to elucidate the magnitude of neutralizing antibody responses against RSV-A and B strains, the specificity and binding characteristics of antibodies to RSV prefusion F and G proteins, antibody affinity maturation, the efficiency of transplacental antibody transfer, and the impact of factors such as gestational age and timing of vaccination during pregnancy. This in-depth analysis provides critical insights into the quality of antibody responses generated following maternal RSV vaccination to help optimize vaccination strategies and inform evidence-based recommendations for maternal RSV immunization programs.

## Results

### Maternal RSV vaccination induces high neutralizing antibodies against RSV-A2 and RSV-B1 in both maternal and cord blood

**Fig. 1:**
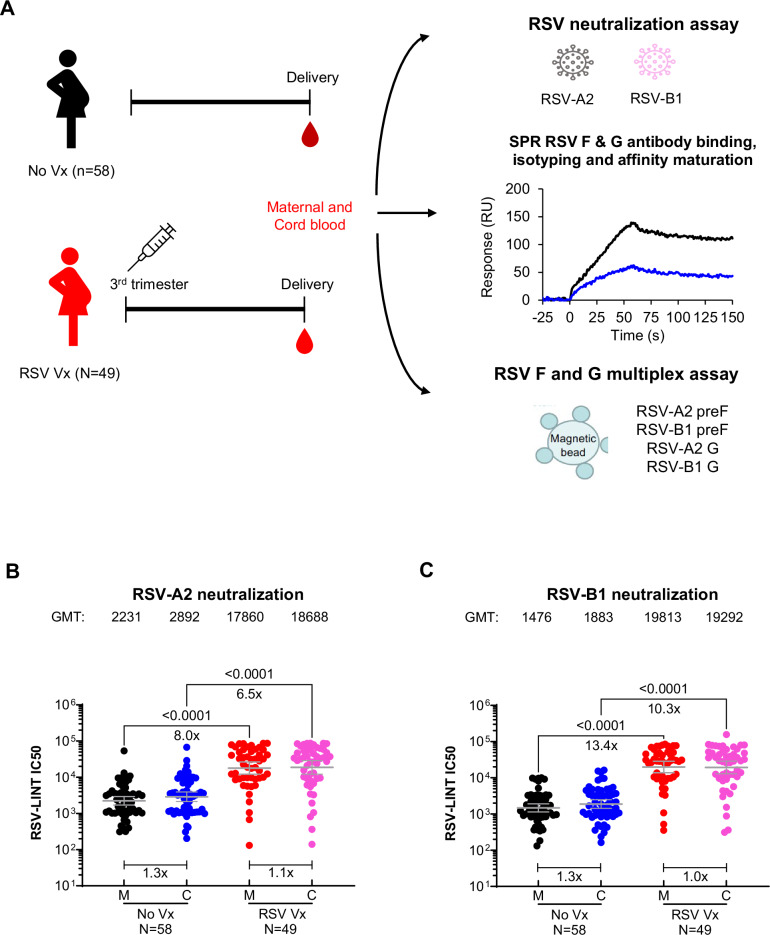
Study design and neutralizing antibodies in mother-infant pairs generated following of maternal RSV vaccination. **A** Schematic design of the maternal RSV vaccine immunization is shown. Samples were collected at delivery from 58 mother-infant pairs in the unvaccinated (No Vx) group and 49 mother-infant pairs in the Abrysvo vaccinated (RSV Vx) group vaccinated with Pfizer RSV vaccine (Abrysvo) given at 32- and 36-weeks’ gestation. Comprehensive antibody profiling of mother and cord blood samples from both cohorts was performed via RSV-A2 and RSV-B1 neutralizing antibodies in the RSV-LINT assay, RSV-A2 prefusion F and G attachment glycoprotein binding antibody kinetics and isotyping using surface plasmon resonance, and IgG interactions with RSV-A2 and RSV-B1 prefusion F and G glycoproteins in a multiplex bead-based assay. **B**, **C** Neutralizing antibody titer (RSV-LINT50; serum titer to reduce 50% RSV infectious units) against RSV-A2 (**B**) and RSV-B1 (**C**) strains are shown for mother (M) and cord blood (C) samples from the unvaccinated group (No Vx; *n* = 58) and in the Abrysvo vaccinated group (RSV Vx; *n* = 49). Geometric means are shown as the central line, with the whiskers indicate 95% confidence interval. GMT values are shown above for each of the groups. The fold change between cord blood vs. mother pairs within each group, as well as between vaccinated vs unvaccinated samples, is shown. Differences between mother-cord blood pairs within each group or corresponding samples between vaccinated and unvaccinated groups were examined for statistical significance by two-sided, paired or unpaired *t* tests, respectively, and statistically significant values (*p*-values < 0.05) are shown.

We evaluated paired maternal-cord blood samples from 107 individuals recruited between July 1, 2023, and June 30, 2024, as part of an ongoing longitudinal cohort study at a large university referral center (Supplementary Table S1). Participants were included in a convenience sample if they had no known fetal genetic anomaly and had paired maternal and cord blood samples. Maternal blood samples were collected within 72 hours of delivery and cord blood samples at delivery; all were processed and stored frozen until analysis. We compared immune responses between 58 unvaccinated control and 49 Abrysvo-vaccinated pregnancies (Fig. 1A). Maternal delivery and cord blood samples were tested to assess antibody transfer and immune profile characteristics. The primary endpoint focused on neutralizing antibody responses against both RSV-A2 and RSV-B1 strains contained in the vaccine using the established RSV- Luciferase Inhibition Neutralization Test (RSV-LINT), with additional quantitative and qualitative analysis of binding antibodies to RSV pre-fusion (preF) and attachment (G) proteins (Fig. 1A).

We observed robust neutralizing antibodies against both RSV-A2 and RSV-B1 strains in individuals following RSV vaccination in pregnancy (Fig. 1B, C). Most unvaccinated adults have detectable levels of RSV-neutralizing antibodies due to the ubiquitous exposure to RSV infections since birth. In maternal samples, vaccinated individuals showed an 8-fold higher neutralizing antibodies against RSV-A2 compared with unvaccinated individuals (RSV-LINT IC50 geometric mean titer [GMT] of 17,860 versus 2231; *p* < *0.0001*) and a 13.4-fold higher titers against RSV-B1 (RSV-LINT IC50 GMT of 19,813 versus 1476; *p* < *0.0001*). Importantly, this enhanced neutralizing capacity was mirrored in cord blood, with cord blood from vaccinated mothers showing higher antibody levels compared with those of unvaccinated controls with 6.5-fold against RSV-A2 (RSV-LINT IC50 GMT from 2892 to 18,688) and 10.3-fold against RSV-B1 (RSV-LINT IC50 GMT from 1883 to 19,292). The cord blood from vaccinated mothers exhibited neutralizing antibody titers that were comparable to maternal levels. Highly significant differences (*p* < *0.0001*) were noted between vaccinated and unvaccinated groups for both maternal and cord blood samples against both viral strains.

### RSV-preF binding antibodies and enhanced immune response in pregnant individuals and their infants following maternal RSV vaccination

**Fig. 2:**
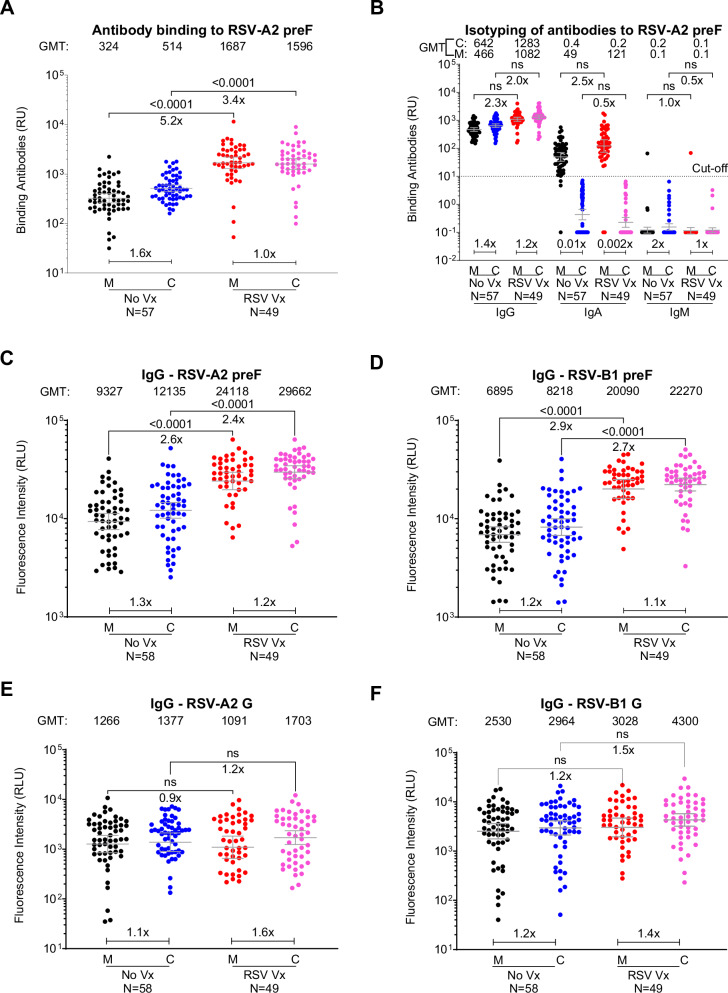
Antibody binding titers and isotyping of mother-infant samples following maternal RSV vaccination against RSV preF and G proteins. **A** Antibody binding of unvaccinated (No Vx; *n* = 57) and post-RSV vaccination (RSV Vx; *n* = 49) maternal (M) and cord blood (C) paired samples to prefusion F (DS-Cav1) of RSV-A2 (RSV preF) measured using SPR is shown as resonance unit (RU) values. **B** Isotyping of human serum antibodies bound to RSV-preF as measured by SPR. The resonance unit for anti-preF bound IgM/IgG/IgA is shown. RU of 10 was defined as the cut-off value for SPR binding antibodies. **C**–**F** Quantification of IgG binding to preF protein of RSV-A2 (**c**) and RSV-B1 (**D**), or RSV-G protein of RSV-A2 (**E**) and RSV-B1 (**F**) with maternal and cord blood samples using bead-based multiplex assay. Each sample from the unvaccinated (No Vx; *n* = 58) and post-RSV vaccination (RSV Vx; *n* = 49) was run in duplicate, and each symbol represents the average fluorescent intensity of duplicate values. The variation for each sample in duplicate runs was < 8%. Geometric means are shown as a central line, with the whiskers indicate 95% confidence interval. GMT values are shown above for each of the groups. Differences between mother-cord blood pairs within each group or corresponding samples between vaccinated and unvaccinated groups were examined for statistical significance by two-sided, paired or unpaired *t* tests, respectively, and statistically significant values (*p*-values < 0.05) are shown.

The analysis of antibody binding characteristics in maternal and cord blood samples revealed significantly higher RSV-specific immune responses following maternal vaccination, particularly in antibodies targeting the prefusion conformation of the RSV fusion proteins contained in the RSV vaccine (Fig. 2). Surface plasmon resonance analysis demonstrated that maternal RSV vaccination induced a substantial 5.2-fold higher (*p* < *0.0001*) antibody binding titer to RSV-preF protein in maternal blood, with cord blood showing a 3.4-fold significantly higher (*p* < *0.0001*) RSV pre-F binding antibody titer compared with unvaccinated controls (Fig. 2A). The GMT for RSV-preF binding antibodies jumped from 324 response units (RU) in maternal blood samples from unvaccinated individuals to 1687 RU in samples from vaccinated individuals, while cord blood GMT levels elevated from 514 RU to 1596 RU respectively. Isotype analysis revealed that the higher preF binding antibody titers in vaccinated individuals was primarily driven by IgG antibodies (2.3-fold higher), especially in cord blood samples (2.0-fold higher) that did not reach statistical significance (Fig. 2B). Higher IgA binding antibodies were observed in maternal blood samples (2.5-fold), but this did not reach statistical significance. No transfer of IgA antibodies from mothers to infants was observed (Fig. 2B).

Multiplex immunoassay analysis provided additional insights into the specificity of the vaccine-induced immune response, confirming the preferential enhancement of anti-preF IgG while showing minimal impact on anti-G protein responses, a protein not included in the RSV vaccine (Fig. 2C–F). The RSV-preF IgG antibody levels were significantly higher (> 2-fold) in both maternal and cord blood samples from vaccinated individuals against both RSV-A2 and RSV-B1 strains, compared with samples from unvaccinated individuals, with geometric mean titers of > 20,000 RLU, demonstrating robust and consistent responses across the vaccinated study population (Fig. 2C, D). The anti-G protein IgG responses remained relatively unchanged following vaccination, suggesting that the bivalent Abrysvo vaccine, which is composed of stabilized prefusion F proteins from RSV A2 and B1, primarily elicits F-specific immune responses rather than broadly enhancing all RSV-specific antibodies (Fig. 2E, F).

### Maternal and infant neutralizing RSV antibody response by gestational age at birth and impact of maternal RSV vaccination

**Fig. 3:**
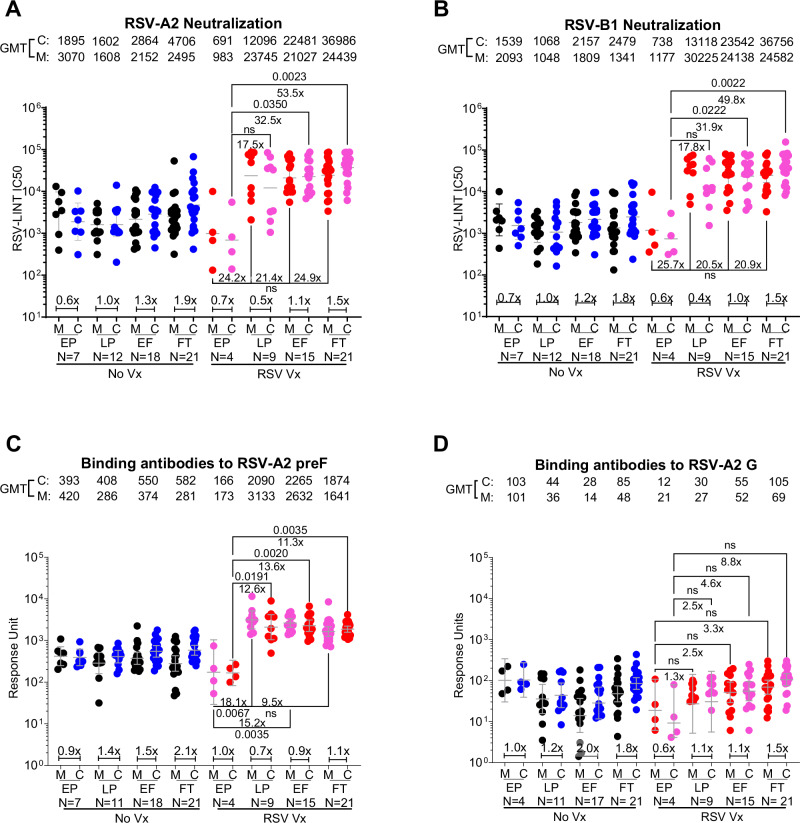
Gestational age at maternal vaccination and timing of delivery impacts RSV maternal-infant antibody response. The two cohorts: unvaccinated group (No Vx; *n* = 58) and in the Abrysvo vaccinated group (RSV Vx; *n* = 49) maternal (M) and cord blood (C) paired samples were categorized by births into four gestational age groups: early preterm (< 34 weeks: EP), late preterm (34–36 weeks; LP), early full term (37-38 weeks; EF), and full term (> 38 weeks; FT), to determine impact of gestational age on maternal antibody transfer efficiency. **A**, **B** Neutralizing antibody titer (RSV-LINT50; serum titer to reduce 50% RSV infectious units) against RSV-A2 (**A**) and RSV-B1 (**B**) strains are shown for maternal (M) and cord blood (C) samples. **C**, **D** Antibody binding of maternal-infant paired samples to RSV-A2 preF (**c**) and RSV-A2 G (**D**) proteins measured using SPR is shown as resonance unit (RU) values. Geometric means are shown as the central line, with the whiskers indicate 95% confidence interval. GMT values are shown above for each of the groups. The fold-change between cord blood vs. maternal blood samples within each group, as well as between vaccinated vs unvaccinated samples, is shown. Differences between maternal-cord blood pairs within each group were examined for statistical significance by two-sided, paired or unpaired* t* tests, respectively, and statistically significant values (*p*-values < 0.05) are shown.

The maternal gestational age at the time of vaccination and its impact on antibody transfer provide critical insights into the impact of timing for maternal RSV vaccination, which may contribute to the vulnerability of preterm infants to RSV disease. An increase in cord blood RSV-specific antibody was observed by increasing gestational age group: from early preterm (< 34 weeks), late preterm (34–36 weeks), early full term (37-38 weeks), and full term (39 + weeks) (Fig. 3). For RSV-A2 neutralizing antibodies, a significantly higher maternal antibodies following vaccination was observed in the late preterm group (24.2-fold) to full term deliveries (24.9-fold), compared with early preterm infants (Fig. 3A). The early pre-term infants showed the lowest enhancement (0.4-fold) compared with unvaccinated group. Similarly, RSV-B1 neutralizing antibodies demonstrated the greatest maternal response in late preterm deliveries (25.7-fold higher) and full-term (20.9-fold), compared with early preterm babies, who showed minimal enhancement (0.5-fold) relative to the unvaccinated group (Fig. 3B).

The cord-to-maternal antibody transfer ratios provided crucial information about the efficiency of transplacental antibody transfer at different gestational age periods. In unvaccinated pregnancies, the cord-to-maternal neutralizing antibody ratio was highest in full-term deliveries (1.9 for RSV-A2 and 1.8 for RSV-B1) but decreased with lower gestational age, reaching 0.6 and 0.7, for RSV-A2 and RSV-B1, respectively, for early pre-term deliveries (Fig. 3A, B). However, following maternal RSV vaccination, this RSV-neutralizing antibody pattern was altered, with reduced neutralization titers for pre-term deliveries. For early pre-term deliveries, the cord titers in the maternal vaccination cohort were 691 and 738, compared with 1,895 and 1539 in the unvaccinated cohort, against RSV-A2 and RSV-B1, respectively. But cord-to-maternal neutralizing antibody ratio were consistent for early and full-term gestational ages, indicating efficient transplacental antibody transfer with cord:maternal neutralization titer ratio of ≥ 1.0, suggesting that the high neutralizing antibody levels achieved through vaccination in pregnant individuals need time for efficient transplacental IgG transfer to the fetus.

Similar cord-to-maternal patterns were observed in the binding antibody analysis for RSV-preF protein, with late preterm deliveries showing enhanced maternal responses, but variable transplacental transfer efficiency in cord blood (Fig. 3C). The RSV-G binding antibody profile was not impacted by maternal RSV preF vaccination. (Fig. 3D).

**Fig. 4:**
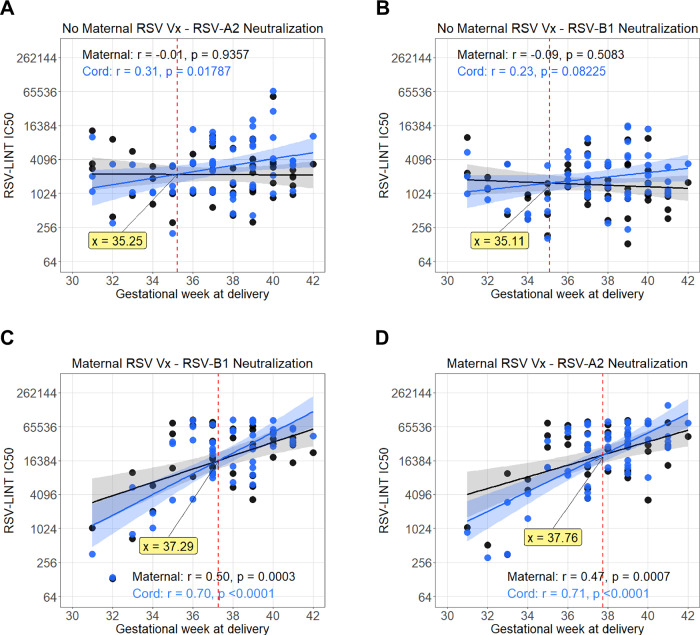
Relationship between gestation age and vaccination on RSV neutralizing antibody response in mothers and infants following maternal RSV vaccination. Correlation between gestation age at delivery and neutralizing antibody titers at birth against RSV-A2 (**A**, **C**) and RSV-B1 (**B**, **D**) in maternal (in black) and cord blood (in blue) samples from the unvaccinated group (*n* = 58; **A**, **B**) and in the Abrysvo vaccinated group (*n* = 49; **C**, **D**). Linear regression (center line) with 95% confidence intervals (shaded) and Spearman correlations for a 2-sided test are shown. The ‘x’ intercept (red dashed lines) denotes the gestational week at delivery when cord:maternal neutralization titer ratio is 1.0.

We further examined the impact of maternal vaccination on the relationship of gestational age at delivery in terms of neutralizing antibody responses (Fig. 4). In unvaccinated pregnancies, an expected gestational age-dependent correlation was observed for neutralizing antibodies in cord blood, with earlier gestational delivery generally associated with lower neutralizing antibody titers against both RSV-A2 and RSV-B1 strains (Fig. 4A, B). Maternal neutralization antibody titers were not impacted by gestational age for both RSV A2 and B1 strains in the unvaccinated cohort. The neutralizing antibodies in these maternal-cord pairs reached equal levels around week 35 of pregnancy for both RSV strains (Fig. 4A, B and Supplementary Fig. 1). This baseline pattern was dramatically altered following maternal vaccination, with vaccinated mothers and cord blood showing moderate to strong correlations between neutralization antibody titers and gestational age at delivery for both RSV strains (Fig. 4C, D). Interestingly, following maternal RSV vaccination, the maternal-cord neutralizing antibodies reached equivalent levels around 37.5 weeks, approximately two weeks later than the unvaccinated cohort (Fig. 4C, D and Supplementary Fig. 1). This comparable cord-to-maternal neutralizing antibody (antibody ratio of one at ~ 37.5 weeks gestational age) was about four weeks post-maternal vaccination, as the mean vaccination gestational age was 33.2 weeks for this pregnancy cohort (Supplementary Table S1).

### Maternal and infant neutralizing RSV antibody by time from vaccination to delivery

**Fig. 5:**
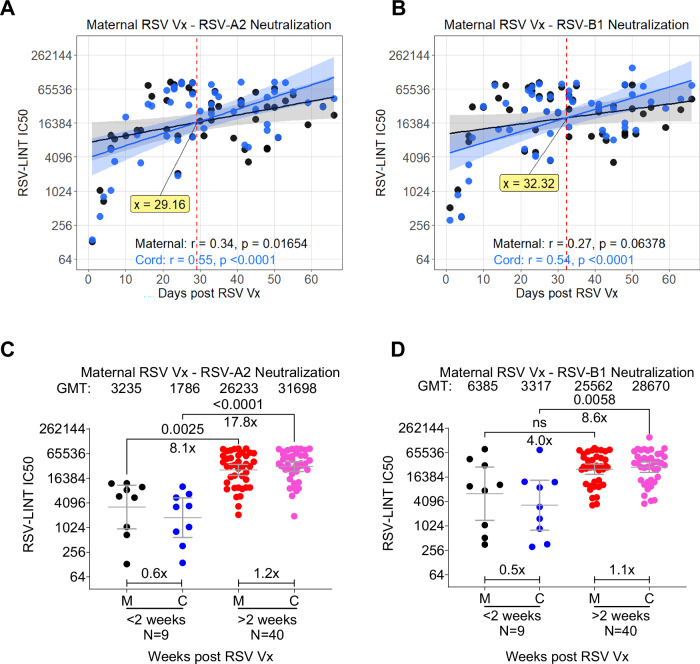
Relationship between vaccination timing on RSV neutralizing antibody response in mothers and infants following maternal RSV vaccination. **A**, **B** Relationship between time-interval since maternal RSV vaccination and maternal-cord RSV neutralizing antibody titers at birth against RSV-A2 (a) and RSV-B1 (**B**) in the Abrysvo vaccinated group (*n* = 49) in maternal (in black) and cord blood (in blue) samples. Linear regression (center line) with 95% confidence intervals (shaded) are shown. Spearman correlations for 2-sided test are given by group. The ‘x’ intercept (red dashed lines) denotes the weeks post-maternal RSV vaccination when cord:maternal neutralization titer ratio is 1.0. **C**, **D** Stratification of neutralizing antibody titers based on time-interval (> 2 weeks vs. ≤ 2 weeks before delivery) between RSV vaccination and birth on maternal-cord neutralizing antibody response against RSV-A2 (**C**) and RSV-B1 (**D**) strains are shown for maternal (M) and cord blood (C) samples from the Abrysvo vaccinated group (RSV Vx;* n* = 49). Geometric means are shown as a central line, with the whiskers indicate 95% confidence interval. GMT values are shown above for each of the groups. The fold-change between maternal-cord pairs within each group, as well as between vaccinated vs unvaccinated samples, is shown. Differences between maternal-cord blood pairs within each group or corresponding samples between vaccinated and unvaccinated groups were examined for statistical significance by two-sample paired or unpaired* t* tests, respectively, and statistically significant values (*p*-values < 0.05) are shown.

The analysis of time intervals between vaccination and delivery provided critical insights into the impact of timing for maternal RSV immunization (Fig. 5). For both RSV A2 and B1 strains, maternal samples showed moderate positive correlation, while cord samples exhibited stronger correlation of neutralizing antibodies with longer time intervals between maternal vaccination and delivery (Fig. 5A, B). Following maternal RSV vaccination, the maternal-cord neutralizing antibodies reached equivalent levels around 29-32 days post-vaccination against both RSV-A2 and RSV-B1 strains (Fig. 5A, B and Supplementary Fig. 2). This coincides with the peak immune response following maternal RSV vaccination, which is approximately a month after vaccination. Sub-group analysis revealed that pregnancies with maternal RSV vaccination occurring more than 2 weeks before delivery showed significantly higher neutralizing antibody responses compared with those vaccinated less than 2 weeks before delivery, with higher impact observed on cord blood (8.6 to 17.8-fold) than for maternal (4 to 8.1-fold) samples, against both RSV strains (Fig. 5C, D).

### Maternal RSV vaccination significantly improves antibody affinity maturation to preF that efficiently transfers to infants

**Fig. 6:**
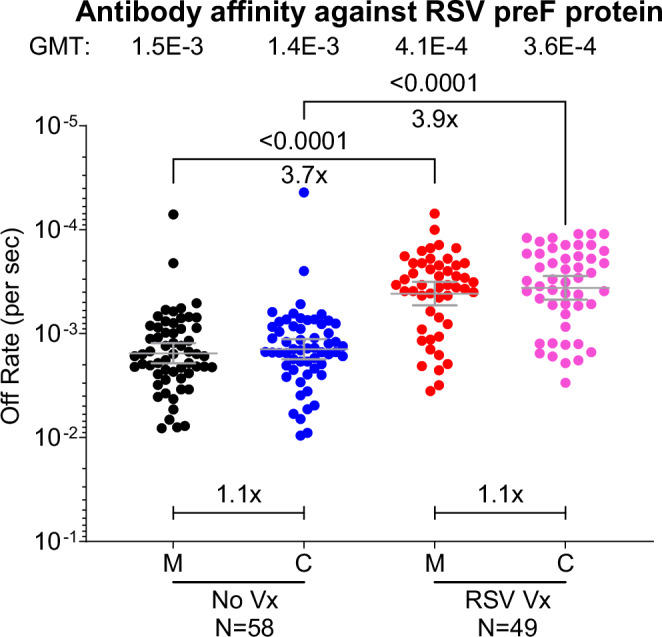
Polyclonal antibody affinity maturation of maternal-cord samples to RSV-A2 preF following maternal RSV vaccination. Antibody affinity of the maternal (M) and cord blood (C) samples was determined by measuring dissociation kinetics (off-rates) of polyclonal antibodies from all individuals against vaccine-homologous prefusion F (DS-Cav1) of RSV-A2 (RSV preF) using SPR. Antibody off-rate constants that describe the fraction of antibody-antigen complexes decaying per second were determined directly for each sample from the unvaccinated (No Vx; *n* = 58) and post-RSV vaccination (RSV Vx; *n* = 49). Each symbol represents the average of duplicate values. The variation for each sample in duplicate runs was < 5%. Means are shown as the central line, with the whiskers indicate 95% confidence interval. GMT values are shown above for each of the groups. The fold change between maternal-cord pairs within each group, as well as between vaccinated vs unvaccinated samples, is shown. Differences between maternal-cord blood pairs within each group or corresponding samples between vaccinated and unvaccinated groups were examined for statistical significance by two-side, paired or unpaired *t* tests, respectively, and statistically significant values (*p*-values < 0.05) are shown.

An important attribute of an effective vaccine is to induce high antibody affinity maturation to the protein target contained in the vaccine. Therefore, we evaluated the impact of maternal RSV vaccination in maternal-cord paired blood samples using real-time kinetics by surface plasmon resonance against the RSV-A2 preF protein. The assessment of antibody affinity maturation revealed a crucial qualitative improvement in the maternal-cord immune response following maternal RSV vaccination, demonstrating that vaccination enhances not only the quantity but also the quality of RSV-specific antibodies (Fig. 6). Surface plasmon resonance analysis of antibody binding kinetics to RSV-A2 prefusion F protein showed that maternal vaccination induced a significant 3.7-fold improvement in antibody affinity in maternal blood and a 3.9-fold improvement in cord blood compared with unvaccinated controls. The affinity measurements, expressed as dissociation constants, decreased from 0.0015 per second in unvaccinated mothers to 0.00041 per second in vaccinated participants, indicating stronger binding interactions between antibodies and the RSV-A2 preF antigen. This improvement in antibody affinity was accompanied by similar enhancements in cord blood samples, with affinity constants improving from 0.0014 to 0.00036 per second, demonstrating that maternal RSV vaccination-induced high-quality antibodies are efficiently transferred across the placenta.

## Discussion

The protective efficacy of maternal RSV vaccination has been established in large randomized controlled trials, demonstrating significant reductions in severe RSV disease in infants^10,11^. The comprehensive analysis of immune profiles following maternal RSV vaccination provides critical mechanistic insights into how this protection is achieved—by the ability of the maternal bivalent RSV preF vaccine (Abrysvo) to generate high-titer, highly affinity-matured neutralizing antibodies that efficiently transfer to the fetus to potentially protect infants during their most vulnerable period of life. The demonstration of 6.5 to 13.4-fold higher neutralizing antibodies against both major RSV subtypes represents a substantial immunological enhancement that aligns well with neutralizing antibody levels reported in the Abrysvo clinical trials^13^ that demonstrated significant reductions in severe RSV disease among infants born to vaccinated women^10,11^. The preferential enhancement of antibodies targeting the RSV prefusion F protein, with minimal impact on attachment G protein responses, documents the mechanism of vaccine-induced protection and validates the scientific rationale behind prefusion F-based vaccine design^6^. This specificity is consistent with structural biology studies demonstrating that the prefusion conformation of the F protein contains the most potent neutralizing epitopes, including sites Ø and V, which are present only in the prefusion conformation, while site II is present in both conformations but is more accessible in the prefusion state^4^. The observation that anti-G protein antibodies remain largely unchanged following vaccination suggests that protection is primarily mediated through F-specific responses, which is advantageous given that F protein is more conserved between RSV subtypes than the G protein^14^. This finding supports the current vaccine design strategy that the observed neutralizing activity is likely to be broadly protective against circulating RSV strains^6^.

This study assessed both the characterization of the quality of RSV antibodies transferred across the placenta as well as demonstrated significant antibody affinity maturation following maternal vaccination. The gestational age-dependent patterns of antibody transfer have profound implications for maternal vaccination strategies and highlight potential reasons for enhanced vulnerability of preterm infants to RSV disease^15^. Transplacental transfer of antibodies, primarily limited to IgG, begins in the second trimester, but increases significantly during pregnancy and in the third trimester, fetal IgG levels often exceeds maternal levels^16,17^. RSV is a common and highly contagious virus, and most children are exposed to RSV by two years of age^18^, and so the adult population has pre-existing antibodies to RSV proteins. The finding that RSV-specific antibody transfer efficiency increases with gestational age, with enhanced transfer occurring after 34 weeks’ gestation, aligns with previous studies of maternal antibody transfer for RSV and other pathogens and reflects the developmental maturation of the placental IgG transport mechanism^17,19,20^. Since early preterm infants are born immediately after or shortly after maternal RSV vaccination, there is reduced time for maternal vaccine response and shorter duration for transplacental antibody transfer, which suggests that current RSV vaccination timing recommendations may not optimally protect this high-risk pre-term population. Our findings suggest that vaccination earlier than 32 weeks, may potentially provide better protection for preterm infants.

The temporal analysis demonstrating the importance of adequate time intervals between vaccination and delivery provides practical guidance for optimizing maternal vaccination programs. Our data suggest that adequate time is required for the development of robust immune responses following vaccination and for efficient transfer of antibodies to the fetus. The finding that vaccinations occurring more than 2 weeks before delivery results in significantly higher antibody concentrations in infants supports current US recommendations for vaccination between 32–36 weeks gestation, as this timing allows for sufficient immune response development while ensuring that most pregnancies will have at least 2 weeks between vaccination and delivery. This is also supported by clinical trials in the United Kingdom, where vaccine effectiveness was substantially higher in infants born to mothers who were vaccinated more than 14 days before delivery^21^. However, the results also suggest that earlier vaccination within this recommended window may be preferable, particularly for pregnancies at risk of preterm delivery, to maximize the duration available for immune response development and antibody transfer. The strong correlations of RSV-specific antibodies in maternal-cord samples observed in the RSV vaccinated cohorts suggest that maternal antibody levels could potentially be used as a predictor of infant protection if a specific level of neutralizing antibody were known. If the safety of maternal vaccines is demonstrated further throughout pregnancy, global maternal immunization programs in countries where dating of gestational age is not routine could benefit from increased timing intervals for receipt of the RSV vaccine. This has particular relevance for pregnancies at risk for preterm delivery, where earlier vaccination could maximize the opportunity for maternal vaccine response and RSV-specific antibody transfer^22^. These findings highlight the particular vulnerability of early preterm infants who may not receive optimal antibody levels due to both reduced maternal immune response and shorter duration for antibody transfer, supporting the potential impact for earlier RSV maternal vaccination timing to maximize protection for this high-risk population.

The demonstration of significant antibody affinity maturation following maternal vaccination represents an important finding that extends our understanding of vaccine-induced immunity beyond simple antibody quantity measurements. Affinity maturation is a hallmark of effective immune responses and occurs through somatic hypermutation and selection processes in germinal centers during the adaptive immune response. Maternal vaccination-induced antibody affinity maturation can potentially be due to two mechanisms: (1) true affinity maturation through somatic hypermutation of existing B-cell clones versus (2) preferential expansion of pre-existing high-affinity B-cell clones. To tease out this important mechanistic point, it would require B-cell receptor sequencing and lineage tracing. The observation that maternal RSV vaccination induces 3.7 to 3.9-fold improvement in antibody affinity in polyclonal sera we observed is substantial and consistent with affinity maturation through somatic hypermutation. Studies of RSV infection in adults and infants have shown much more modest affinity improvements^23,24^, suggesting that simple clonal expansion without SHM would not achieve this magnitude of improvement. This indicates that RSV vaccination recalls circulating memory B cells in these RSV-experienced adults that are likely to re-enter secondary germinal centers to undergo further somatic hypermutation (SHM) and antibody affinity maturation and generate antibodies with enhanced antigen recognition and functional capacity^25^. The observed polyclonal antibody affinity against RSV-preF is at the high-end of the polyclonal antibody affinity (off-rate of 10^−4^ per sec) observed in polyclonal samples following vaccination with several viral vaccines^26–28^. The vaccine induced antibody affinity maturation was much higher than those observed following RSV infection either in adults or infants measured by the SPR-based assay or by analyzing somatic hypermutation^23,24^. The significance of improved antibody affinity extends beyond simple binding strength, as higher-affinity antibodies typically provide more effective and longer-lasting protection. In previous studies, we had demonstrated a strong correlation between antibody affinity and protection from highly pathogenic avian influenza viruses^29,30^ and a correlation with clinical benefit in patients infected with Zika virus^31^, Ebola virus^32^, influenza virus^33^ and COVID-19^34,35^. The efficient transfer of high-affinity maternal antibodies to the fetus suggests that infants receive not only large quantities of protective antibodies but also antibodies of superior quality, which may translate to enhanced clinical protection. This finding has important implications for the duration and effectiveness of passive immunity transferred to infants, as higher-affinity antibodies are generally more effective at neutralizing viral infection and may provide protection at lower concentrations. The statistical significance (*p* < *0.0001*) of the affinity improvements in both maternal and cord blood samples underscores the consistent and substantial qualitative enhancement of the immune response achieved through maternal RSV vaccination.

Study limitations include the lack of long-term longitudinal follow-up in our population to determine the clinical outcome in terms of RSV infection rates or severity in the infants. We were unable to determine the duration of protection provided by maternally-derived antibodies in the context of maternal RSV immunization. Moreover, BCR sequencing of RSV-F + B cell populations after maternal immunization would be helpful to confirm and delineate the mechanism of antibody affinity maturation observed in serum samples. Investigation of specifically designed studies to evaluate earlier vaccination timing for pregnancies at risk for preterm delivery, as suggested by the current findings, could lead to refined recommendations to better protect the most vulnerable infant populations. Finally, real-world effectiveness studies in diverse populations and healthcare settings will be crucial for validating the clinical impact and safety of maternal RSV vaccination programs and informing global implementation strategies.

The implications of this research extend beyond immediate clinical applications to inform future vaccine development and optimization strategies for maternal immunization. The detailed characterization of antibody responses provides benchmarks for evaluating next-generation RSV vaccines and may guide the development of improved formulations or alternative delivery strategies. The finding that prefusion F-based vaccines can induce robust affinity maturation suggests that similar approaches might be successful for other pathogens where maternal vaccination may provide infant immune protection^36^. In addition, the gestational age-dependent patterns of antibody transfer may inform vaccination strategies for other maternal vaccines and contribute to our broader understanding of maternal-fetal immunology^19^.

From a global public health perspective, these findings support the potential for maternal RSV vaccination to provide high levels of quality antibodies against RSV to protect infants during early life. The robust immune responses observed across different gestational ages and the efficient transfer of high-quality antibodies suggest that maternal vaccination could be particularly beneficial in countries where RSV mortality rates are highest, and access to intensive care for severe RSV disease may be limited. The demonstration that a single maternal vaccination can generate high antibody levels to RSV, combined with the safety profile established in clinical trials, supports the implementation of maternal RSV vaccination in routine prenatal care.

## Methods

### Study samples

Study participants enrolled in a prospective cohort study investigating maternal immunity in low- and high-risk pregnancies at the University of Washington (UW) provided maternal and cord blood samples for this study. Inclusion criteria for participation included the ability to obtain informed consent and the availability of paired maternal-cord blood samples obtained between 7/1/2023 and 6/30/2024. This study received ethics approval through the UW Human Subjects Division. All study participants provided written informed consent. Permission to test de-identified samples was obtained from the U.S. FDA Research Involving Human Subjects Committee (FDA-RIHSC) under exemption protocol #12-079B.

Based on the CDC definition, we categorized births into four gestational age groups: early preterm (< 34 weeks), late preterm (34–36 weeks), early full term (37-38 weeks), and full term (> 38 weeks), allowing for detailed examination of how maternal antibody transfer efficiency varies with gestational age. For inclusion in the vaccinated cohort, participants must have received the RSV vaccine at least 24 h prior to delivery to allow for an initial immune response. Two participants who delivered within 24 h of vaccination were excluded from the vaccinated cohort analyses.

### Cells, viruses, and proteins

A549 cells (Cat. No. #CCL-185) were obtained from the American Type Culture Collection (ATCC, Manassas, VA, USA) and were maintained in F-12K medium supplemented with 10% fetal bovine serum, 1X penicillin streptomycin (P-S), and L-glutamine. RSV-A2-Renilla Luciferase (RSV-A2-Rluc) or RSV-B1 expressing renilla luciferase (RSV-B1-Rluc), which expresses the renilla luciferase gene upstream of the NS1 gene, was prepared by infecting sub-confluent A549 cell monolayers, as described before^37^. The pre-fusion RSV F (DS-Cav1) plasmid encoding amino acid residues 26-513Δ110-136 of the RSV-A2 strain was a kind gift from Peter Kwong (VRC, NIH). DS-Cav1 protein was produced in 293 F cells as described before^6^. Pre-fusion RSV-F protein of RSV-B1 strain was obtained from Sino Biologicals. Recombinant ectodomain coding sequence (67–298) of RSV A2 G or RSV-B1 strain was produced using mammalian 293-Flp-In cells to obtain fully glycosylated RSV-A2 G (RMG-A2) or RSV-B1 G (RMG-B1) as described previously^38^.

### RSV- Luciferase Inhibition Neutralization Test (RSV-LINT)

Samples were analyzed for neutralizing antibodies against RSV-A2 and RSV-B1 strains in a qualified RSV-LINT as described before^37^. Briefly, A549 cells were seeded at 2 × 10^4^ cells in 96-well plates. Heat-inactivated serum samples were serially diluted 2-fold from 1:10 to 1:2560, added to RSV-A2-Rluc or RSV-B1-Rluc virus (final serum dilution of 1:20 to 1:5120), and incubated at 37 ^o^C for 1 h. For samples demonstrating > 90% neutralization at the highest initial dilution tested, extended 3-fold dilution series (up to 1:87,480) were performed to accurately determine IC50 values. The luciferase assay was performed as described previously^37^. Each serum dilution was run in duplicate, with appropriate positive and negative controls.

Luminescent readings for test samples were normalized using the virus only (no serum added) control values and multiplied by 100 to obtain the percent of control. The percentage of control values were used for linear regression analyses using log10 of the reciprocal of the serum dilution on the X-axis. The trendline of the linear part of the curve was used to calculate the slope and y-intercept and 50% inhibition endpoint titers (RSV-LINT IC50) calculated using the formula: antilog of [(50 + y-intercept)/slope]. If no neutralization was detected, a titer of 1 was assigned to the samples.

Our Laboratory participated in the interlaboratory studies that assessed the 1st World Health Organization (WHO) International Standard for Antiserum to RSV (16/284; NIBSC) in our neutralization assays for RSV-A^39^ and RSV-B^40^. To approximate WHO International Units/mL, our RSV-LINT IC50 neutralization titers may be multiplied by the conversion factor of 0.382 for RSV-A2 and 0.373 for RSV-B1.

### Surface plasmon resonance (SPR)

Steady-state equilibrium binding of polyclonal antibodies in maternal serum and cold blood samples was monitored at 25 °C using a ProteOn surface plasmon resonance biosensor (BioRad) as previously described^23,41,42^. Briefly, the purified recombinant RSV-A2 preF (DS-Cav1) and RSV-A2 G protein were coupled to a GLC sensor chip via amine coupling with 200 resonance units (RU) in the test flow channels, and the spatial density of the proteins was optimized such that there was a single monovalent interaction for each antibody molecule. SPR assay measures the binding of all antibody isotypes. Samples of 300 µL freshly prepared 10-, 30-, and 100-fold dilutions were injected at a flow rate of 50 µL/min (120 sec contact duration) for association, and disassociation was performed over a 1200 s interval. Responses from the protein surface were corrected for the response from a mock surface and for responses from a buffer-only injection. Total antibody binding and data analysis results were calculated with BioRad ProteOn manager software (version 3.0.1).

Antibody off-rate constants, which describe the fraction of antigen-antibody complexes that decay per second, were determined directly from the sample interaction with F or G proteins using SPR in the dissociation phase using only sensorgrams with Max RU in the range of 10-150 RU and calculated using the BioRad ProteOn manager software for the heterogeneous sample model as described before. Off-rate constants were determined from two independent SPR runs^27,43^.

### IgG binding to F and G proteins for RSV-A2 and RSV-B1 strains in multiplex assay

Samples were analyzed for IgG binding to prefusion F and G proteins of RSV-A2 and RSV-B1 strains with a fluorescent magnetic bead immunoassay. The assay panel consisted of uniquely identifiable Luminex MagPlex Microspheres conjugated to RSV-A2 preF, RSV-B1 preF, RMG-A2, RMG-B1, and control human serum albumin (HSA) protein, using the Bio-Rad amine coupling kit. Following confirmation of coupling, the multiplex immunoassay was performed on the samples, as described previously^44^.

Briefly, an equal number of beads for each unique protein conjugated microsphere were added in a tube and mixed. Samples were thawed and immediately spun down at 7000 × *g* for 1 min, serum was collected, diluted 1:500, and added to a dilution plate. The equal volumes of bead-mix and serum dilutions were combined resulting in final serum concentrations of 1000-fold dilution in 96-well plates. Following incubation, the plates were washed, and PE-labeled secondary anti-human IgG antibody was added. The bead containing plates were then washed followed by reading using a BD LSR Fortessa X-20 cell analyzer, and fluorescent intensity (FI) was collected by BD FACSDiva software.

The fluorescent intensity data were normalized to subtract the blank (FI in the absence of a serum sample) and the FI of each corresponding serum binding to the control HSA protein.

### Statistical analyses

Participant and pregnancy characteristics were described using totals and percentages or means and standard deviations. Student’s *T* tests and Fisher’s exact tests were used to compare continuous variables and categorical variables, respectively, between vaccinated and unvaccinated participants as shown in Supplementary Table S1. Descriptive statistics were performed to determine the geometric mean titer values and were calculated using GraphPad Prism. All experimental data to compare differences between maternal-cord blood pairs within each group or corresponding samples between vaccinated and unvaccinated groups were examined for statistical significance by a two-sided t-test or by ANOVA using the Kruskal-Wallis test for multiple comparisons, respectively, calculated in GraphPad Prism (10.0.02 version) and R version 4.5.0. Correlation coefficients were calculated using Spearman’s method and compared between the two groups using Fisher’s z-transformation. *p*-values less than 0.05 were considered significant with a 95% confidence interval.

### Ethics statement

The study at CBER, FDA, was conducted with de-identified samples under Research Involving Human Subjects (RIHSC) exemption 12-079B, and all assays performed fell within the permissible usages in the original informed consent.

### Reporting summary

Further information on research design is available in the Nature Portfolio Reporting Summary linked to this article.

## Supplementary information

Supplementary Information

Reporting Summary

Transparent Peer Review file

## Source data

Source Data

## Data Availability

All data are shown in the manuscript figures. Source data are provided in this paper.

## Code availability

No custom codes were used in this study.
