# Supplementary material for: Maternal RSV vaccination generates high-affinity antibodies that efficiently transfer to infants, providing enhanced passive immunity
Source: Nat Commun. 2026 May 8;17:6223. doi: 10.1038/s41467-026-72659-3 (PMC13369169; doi:10.1038/s41467-026-72659-3)
Supplement: Supplementary file 1 — Supplementary Information [file 41467_2026_72659_MOESM1_ESM.pdf]

Supplementary Table S1. Participant and pregnancy characteristics

	Total (n=109)	Maternal RSV vaccine (n=51)	No RSV vaccine (n=58)	p-value
Year of delivery				0.57
2023	59 (54.1)	26 (51.0)	33 (56.9)	
2024	50 (45.9)	25 (49.0)	23 (43.1)	
Maternal age	35.0 (4.5)	35.4 (4.3)	34.7 (4.7)	0.41
Race				0.30
Asian	16 (14.7)	11 (21.6)	5 (8.6)	
Black or African American	5 (4.6)	3 (5.9)	2 (3.4)	
Native Hawaiian/Pacific Islander	1 (0.9)	0 (0)	1 (1.7)	
White	78 (71.6)	34 (66.7)	44 (75.9)	
Other	1 (0.9)	0 (0)	1 (1.7)	
Prefer not to answer	8 (7.3)	3 (5.9)	5 (8.6)	
Ethnicity				1.0
Hispanic, Latino, or Spanish origin	10 (9.2)	5 (9.8)	5 (8.6)	
Prefer not to answer	2 (1.8)	1 (2.0)	1 (1.7)	
Insurance status				0.47
Public	12 (11.0)	4 (7.8)	8 (13.8)	
Commercial	94 (86.2)	45 (88.2)	49 (84.5)	
Tricare/Federal	3 (2.8)	2 (3.9)	1 (1.7)	
BMI	30.9 (5.9)	31.1 (5.7)	30.65 (6.1)	0.68
Pregestational DM	4 (3.7)	2 (3.9)	2 (3.4)	1.0
Preeclampsia	17 (15.6)	8 (15.7)	9 (15.5)	0.98
Chronic HTN	13 (11.9)	7 (13.7)	6 (10.3)	0.77
RSV vaccine to delivery (weeks)	4.3 (2.5)	4.3 (2.5)	NA	NA
GA at RSV vaccine dose	33.2 (1.4)	33.2 (1.4)	NA	NA
Delivery gestational age	37.4 (2.7)	37.5 (2.5)	37.2 (2.8)	0.57
Range	30.9 - 41.7	30.9 - 41.7	30.9 - 41.6	
Delivery < 37 weeks	39 (35.8)	17 (33.3)	22 (37.9)	0.69
Delivery ≥ 37 weeks	70 (64.2)	34 (66.7)	36 (62.1)	
Birth weight (grams)	2946.4 (873.67)	3042.0 (916.3)	2862.3 (833.3)	0.29
Infant Sex				0.847
Female	60 (55.0)	29 (56.9)	31 (53.4)	
Male	49 (45.0)	22 (43.1)	27 (46.6)	
Neonatal NICU admission	37 (33.9)	18 (35.3)	19 (32.8)	0.84

Notes: mean (SD) or N (%); continuous variables compared using t-test and categorical variables compared using Fisher's exact tests

Abbreviations: BMI = body mass index; DM = diabetes mellitus; HTN = hypertension; GA = gestational age; NICU = neonatal intensive care unit

Supplementary Figure 1

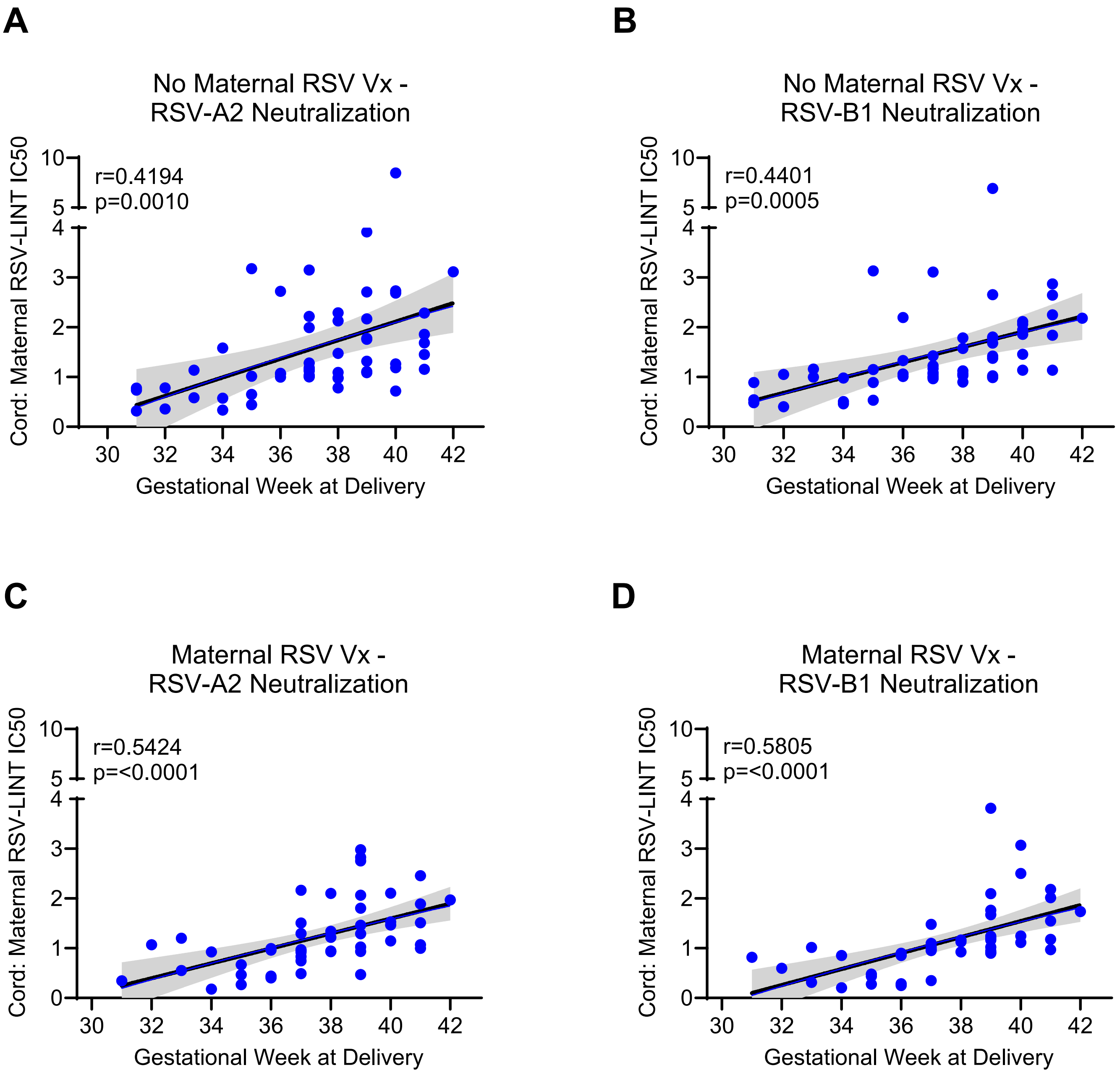

Supplementary Figure 1. Relationship between ratio of RSV neutralizing antibody response in infants and mothers with gestational age following maternal RSV vaccination.
Correlation between gestation age at delivery and cord:maternal neutralization titer ratio at birth against RSV-A2 (a & c) and RSV-B1 (b & d) in samples from the unvaccinated group (n=58; a-b) and in the Abrysvo vaccinated group (n=49; c-d). Linear regression with 95% confidence intervals (shaded), with Spearman correlations and significant values (p values <0.05) are shown.

Supplementary Figure 2

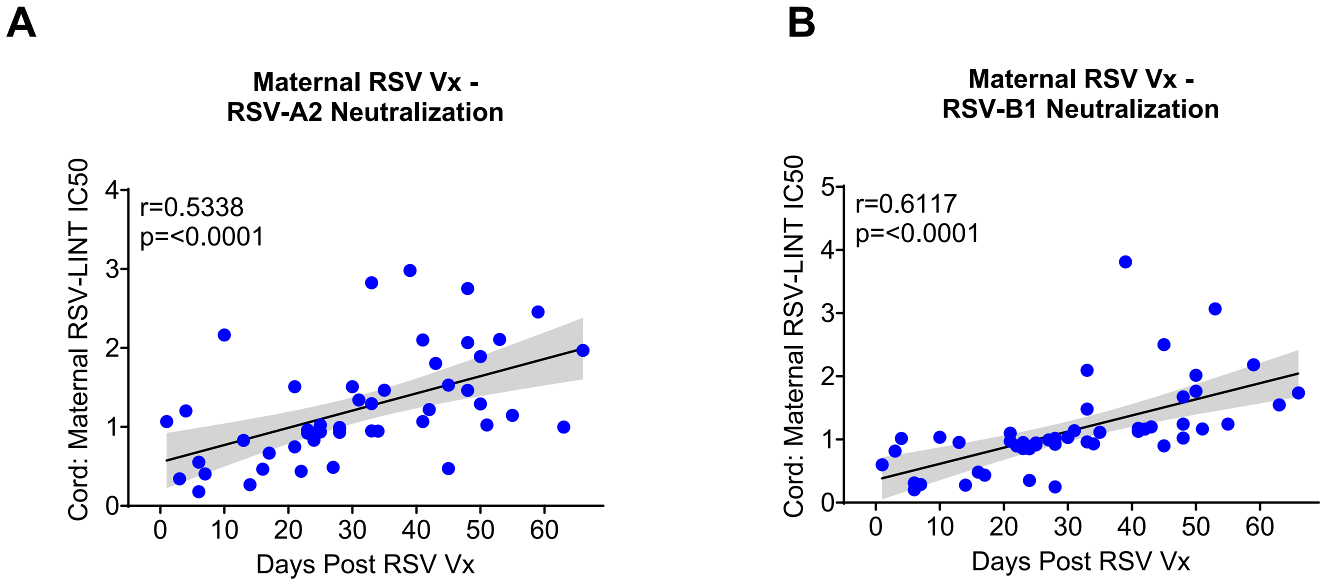

Supplementary Figure 2. Relationship between vaccination timing on cord:maternal RSV neutralization titer ratio following maternal RSV vaccination.
Correlation between time-interval since maternal RSV vaccination and maternal-cord RSV neutralizing antibody titers at birth against RSV-A2 (a) and RSV-B1 (b) in the Abrysvo vaccinated group (n=49) samples. Linear regression with 95% confidence intervals (shaded), with Spearman correlations and significant values (p values <0.05) are shown.
