# Supplementary material for: Maternal RSV vaccination generates high-affinity antibodies that efficiently transfer to infants, providing enhanced passive immunity
Source: Nat Commun. 2026 May 8;17:6223. doi: 10.1038/s41467-026-72659-3 (PMC13369169; doi:10.1038/s41467-026-72659-3)
Supplement: Supplementary file 2 — Reporting Summary [file 41467_2026_72659_MOESM2_ESM.pdf]

nature portfolio

Corresponding author(s): Surender Khurana

Last updated by author(s): Apr 13, 2026

Reporting Summary

Nature Portfolio wishes to improve the reproducibility of the work that we publish. This form provides structure for consistency and transparency in reporting. For further information on Nature Portfolio policies, see our Editorial Policies and the Editorial Policy Checklist.

Statistics

For all statistical analyses, confirm that the following items are present in the figure legend, table legend, main text, or Methods section.

n/a	Confirmed	
☐	☒	The exact sample size (n) for each experimental group/condition, given as a discrete number and unit of measurement
☐	☒	A statement on whether measurements were taken from distinct samples or whether the same sample was measured repeatedly
☐	☒	The statistical test(s) used AND whether they are one- or two-sided<br>*Only common tests should be described solely by name; describe more complex techniques in the Methods section.*
☐	☒	A description of all covariates tested
☐	☒	A description of any assumptions or corrections, such as tests of normality and adjustment for multiple comparisons
☐	☒	A full description of the statistical parameters including central tendency (e.g. means) or other basic estimates (e.g. regression coefficient) AND variation (e.g. standard deviation) or associated estimates of uncertainty (e.g. confidence intervals)
☐	☒	For null hypothesis testing, the test statistic (e.g. F, t, r) with confidence intervals, effect sizes, degrees of freedom and P value noted<br>*Give P values as exact values whenever suitable.*
☒	☐	For Bayesian analysis, information on the choice of priors and Markov chain Monte Carlo settings
☒	☐	For hierarchical and complex designs, identification of the appropriate level for tests and full reporting of outcomes
☐	☒	Estimates of effect sizes (e.g. Cohen's d, Pearson's r), indicating how they were calculated

Our web collection on statistics for biologists contains articles on many of the points above.

Software and code

Policy information about availability of computer code

Data collection	Antibody data was collected in MS Excel version 16.5.7. BioRad ProteON Manager software (Version 3.1.0) was used to collect antibody binding data from SPR machine (www.Biorad.com)
Data analysis	Participant and pregnancy characteristics were described using totals and percentages or means and standard deviations. Student's T-tests and Fisher's exact tests were used to compare continuous variables and categorical variables, respectively, between vaccinated and unvaccinated participants. Antibody titers were calculated using Prism 9.3.1 (GraphPad Software). BioRad ProteON Manager software (Version 3.1.0) for antibody binding analysis from SPR machine (www.Biorad.com). Statistical analysis were performed using KGraphPad Prism (9.3.1 version) and and R version 4.5.0. Correlation coefficients were calculated using the Pearson's method and compared between the two groups using a Fisher's z-transformation.

For manuscripts utilizing custom algorithms or software that are central to the research but not yet described in published literature, software must be made available to editors and reviewers. We strongly encourage code deposition in a community repository (e.g. GitHub). See the Nature Portfolio guidelines for submitting code & software for further information.

Data

Policy information about availability of data

All manuscripts must include a data availability statement. This statement should provide the following information, where applicable:

- Accession codes, unique identifiers, or web links for publicly available datasets
- A description of any restrictions on data availability
- For clinical datasets or third party data, please ensure that the statement adheres to our policy

All data are shown in the manuscript figures. Source data are provided with this paper in Source data file.

Research involving human participants, their data, or biological material

Policy information about studies with human participants or human data. See also policy information about sex, gender (identity/presentation), and sexual orientation and race, ethnicity and racism.

Reporting on sex and gender	All pregnant women were eligible for the study. The consent was not obtained for reporting and sharing individual-level data. No sex-based analyses were performed in this study.
Reporting on race, ethnicity, or other socially relevant groupings	All pregnant women irrespective of their race or ethnicity were eligible for the study. Race or ethnicity was not considered in the study design and study findings apply to women of all race or ethnicities. No race-based analyses were performed in this study.
Population characteristics	Participants in this study were pregnant women. All pregnant women irrespective of race, ethnicity, or previous health status who provided informed consent were eligible for the different clinical study as described in materials and methods. Race, ethnicity, or previous health status was not considered in the study design and study findings apply to all women of any race or ethnicity. No race-based analyses were performed in this study.
Recruitment	All pregnant women were eligible without any specific selection criteria and no selection bias or any other apparent bias. Samples were collected from pregnant women following informed consent to participate in the clinical trials. Pregnant women participants were recruited and enrolled in the study as per IRB approval.
Ethics oversight	Study participants enrolled in a prospective cohort study investigating maternal immunity in low- and high-risk pregnancies at the University of Washington (UW) provided maternal and cord blood samples for this study. This study received ethics approval through the UW Human Subjects Division.<br>The left-over archived convenience samples that were deidentified were tested blinded in different antibody assays with approval from the U.S. Food and Drug Administration's Research Involving Human Subjects Committee (FDA-RIHSC) under exemption protocol #12-079B.

Note that full information on the approval of the study protocol must also be provided in the manuscript.

Field-specific reporting

Please select the one below that is the best fit for your research. If you are not sure, read the appropriate sections before making your selection.

☒ Life sciences ☐ Behavioural & social sciences ☐ Ecological, evolutionary & environmental sciences

For a reference copy of the document with all sections, see nature.com/documents/nr-reporting-summary-flat.pdf

Life sciences study design

All studies must disclose on these points even when the disclosure is negative.

Sample size	All available samples were analyzed in this study
Data exclusions	No data was excluded
Replication	Neutralization, SPR and multiplex binding experiments were performed twice on each sample by independent researchers in the lab. The replications were successful. The variation in duplicate experimental runs was less than 3-fold (one-serial dilution) for neutralization. The variation in duplicate SPR experimental runs was <5%, and <8% for multiplex assay.
Randomization	All samples from the women were analyzed in this study. The clinical study was blinded and randomized performed in pregnant women. Initially, no patient information was provided, and all the immune analyses were conducted blindly by the researcher's performing the assays.
Blinding	Experiments were performed by different investigators, who were blinded to sample identity.

Reporting for specific materials, systems and methods

We require information from authors about some types of materials, experimental systems and methods used in many studies. Here, indicate whether each material, system or method listed is relevant to your study. If you are not sure if a list item applies to your research, read the appropriate section before selecting a response.

Materials & experimental systems

n/a	Involved in the study	
☐	☒	Antibodies
☐	☒	Eukaryotic cell lines
☒	☐	Palaeontology and archaeology
☒	☐	Animals and other organisms
☒	☐	Clinical data
☒	☐	Dual use research of concern
☒	☐	Plants

Methods

n/a	Involved in the study	
☒	☐	ChIP-seq
☒	☐	Flow cytometry
☒	☐	MRI-based neuroimaging

Antibodies

Antibodies used	Donkey anti-human IgG-Fc specific antibody (Cat # 709-005-098), Donkey anti-human IgM Fc5u specific (Cat # 709-005-073), goat anti-human serum IgA alpha chain specific (Cat # 109-005-011) were purchased from Jackson Immuno Reseearch.
Validation	Isotyping antibodies from Jackson Immuno Reseearch were produced, tested and validated by the manufacturer.

Eukaryotic cell lines

Policy information about cell lines and Sex and Gender in Research

Cell line source(s)	A549 cells (Cat. No. #CCL-185) were obtained from the American Type Culture Collection (ATCC, Manassas, VA, USA) .
Authentication	The cell line was authenticated by karyotyping or other genomic techniques by ATCC.
Mycoplasma contamination	Negative for Mycoplasma
Commonly misidentified lines (See ICLAC register)	No misidentified cell lines were used in the study.
